# Health effects of agrochemicals among farm workers in commercial farms of Kwekwe district, Zimbabwe

**DOI:** 10.4314/pamj.v9i1.71201

**Published:** 2011-07-11

**Authors:** Regis Magauzi, Bigboy Mabaera, Simbarashe Rusakaniko, Anderson Chimusoro, Nqobile Ndlovu, Mufuta Tshimanga, Gerald Shambira, Addmore Chadambuka, Notion Gombe

**Affiliations:** 1 Dept of Community Medicine University of Zimbabwe, P.O. Box A178 Avondale, Harare, Zimbabwe; 2 Provincial Medical Directorate, Midlands Province, P.O. Box 206, Gweru, Zimbabwe

**Keywords:** Organophosphates, cholinesterase activity, pesticide poisoning, agrochemicals, farm workers

## Abstract

**Introduction:**

Farm workers are at a very high risk of occupational diseases due to exposure to pesticides resulting from inadequate education, training and safety systems. The farm worker spends a lot of time exposed to these harmful agrochemicals. Numerous acute cases with symptoms typical of agrochemical exposure were reported from the commercial farms. We assessed the health effects of agrochemicals in farm workers in commercial farms of Kwekwe District (Zimbabwe), in 2006.

**Methods:**

An analytical cross sectional study was conducted amongst a sample of 246 farm workers who handled agrochemicals when discharging their duties in the commercial farms. Plasma cholinesterase activity in blood specimens obtained from farm workers was measured using spectrophotometry to establish levels of poisoning by organophosphate and/or carbamates. Information on the knowledge, attitudes and practices of farm workers on agrochemicals use was collected using a pre-tested interviewer administered questionnaire. Bivariate and multivariate analyses were conducted to determine factors that were associated with abnormal cholinesterase activity.

**Results:**

The prevalence of organophosphate poisoning, indicated by cholinesterase activity of 75% or less, was 24.1%. The median period of exposure to agrochemicals was 3 years (Q_1_:=1 year, Q_3_:=7 years). Ninety eight (41.5%) farm workers knew the triangle colour code for the most dangerous agrochemicals. Not being provided with personal protective equipment (OR 2.00; 95% CI: 1.07 – 3.68) and lack of knowledge of the triangle colour code for most dangerous agrochemicals (OR 2.02; 95% CI: 1.02 – 4.03) were significantly associated with abnormal cholinesterase activity.

**Conclusion:**

There was organophosphate poisoning in the commercial farms. Factors that were significantly associated with the poisoning were lack of protective clothing and lack of knowledge of the triangle colour code for most dangerous agrochemicals. We recommended intensive health education and training of farm workers on the use of agrochemicals, provision of adequate and proper personal protective equipment as mitigation measures to this problem.

## Introduction

Agriculture mortality rates have remained consistently high throughout the world in the last decade in contrast to other dangerous occupations [1]. Farm workers are at a very high risk of occupational diseases due to exposure to pesticides resulting from inadequate education, training and safety systems. In developed countries such as the US, farmers and farm workers comprise only 3% of the workforce, but they account for as much as 8% of all work-related accidents [2]. Developing countries are known to consume less than 20% of the world production of agrochemicals, which are responsible for as much as 1.1 million (70%) of the total cases of acute poisoning in the working population [3].

Zimbabwe is one of the developing countries whose economy is mainly based on agriculture and in order to make foodstuffs of high quality and quantity, extensive use of agrochemicals is implemented [4]. In view of this, the pivot of production is the farm worker who spends a lot of time exposed to these harmful agrochemicals.

Agrochemicals are known to find their way in the blood systems of human beings through the mouth, nose, intact skin and the eyes. Several adverse health effects are known to result from exposure to pesticides including temporary acute effects like irritation of eyes and excessive salivation as well as chronic diseases like cancer, reproductive and developmental disorders. Effects on the Central Nervous System (CNS) like restlessness, loss of memory, convulsions and coma are also common. In addition, effects on parasympathetic and sympathetic nervous system have been widely reported including respiratory paralysis which is fatal [3].

More than 25% of the population of Zimbabwe is in commercial farming areas and with the current land reform programme, the figures are increasing. Although no official statistics regarding chemical poisoning have been reported in Zimbabwe, researchers have established through biological monitoring that prevalence due to agrochemical poisoning is as high as 30% [4]. With the current resettlement programme the figure is likely to be higher than before.

Kwekwe District is in the Midlands Province of Zimbabwe and has approximately 291 large and small scale commercial farms. The use of agrochemicals in these farms is rampant. The district repeatedly reported high incidence (26%) of acute cases like excessive irritation, salivation, diarrhoea and fever which are typical symptoms that are associated with agrochemical exposure and were coming mainly from its commercial farming areas [5]. This study was conducted among farm workers in the commercial farms of Kwekwe District to establish the relationship between exposure to agrochemicals and diseases that were being reported in the district.

## Methods

An analytical cross sectional study was conducted in Kwekwe District commercial farms among farm workers who handle agrochemicals when discharging their duties. Eleven farms were randomly selected using the lottery method from 30 farms in the southern part of the district. We excluded farms in the northern areas where indoor residual spraying for malaria control was conducted annually to avoid chemical poisoning due to malaria vector control.

For our sample size we assumed 95% confidence level and expected prevalence of pesticide exposure (p) of 20% [6] and absolute precision (d) of 5% using the formulae by Dobson [7]:

The required sample n=z^2^ × 246 × (p × (1−p)/d^2^)

Stratified sampling was conducted from one farm to the next until the required sample size was achieved. At each farm, farm workers whose duties involved contact with agrochemicals were categorized into the seven types of exposures that were identified namely, spraying, mixing, stores management, loading, repairing spraying equipment, working in recently sprayed areas and waste management of the agro-chemicals. A sampling fraction at each farm was calculated based on the numbers of farm workers in the types of exposures studied. This sampling method was conducted from one farm to the next until the required sample of 246 participants was achieved. These were then interviewed and 1µl of blood samples was collected from those who consented to the required blood test.

Plasma cholinesterase activity in blood specimens obtained from farm workers was measured using spectrophotometry to establish levels of poisoning by organophosphate and/or carbamates. A mixture of blood, indicator (bromothymol blue) and acetylcholine perchlorate was prepared and allowed to stand for 10-15 minutes. The change in pH during this time period was measured by comparing the colour of the mixture with a set of permanent coloured glass standards contained in a disc. The change of pH was a measure of the level of cholinesterase activity in the blood. Harmful exposure to agrochemicals was defined as blood cholinesterase activity levels of 75% or less [8,9]. A blood sample drawn from the principal investigator (PI) and was tested for cholinesterase activity had 100% cholinesterase activity. This was used as the control result.

The design of the questionnaire was guided by the Health Belief Model (HBM) [10]. All the constructs except self efficacy were measured in this study. We pilot tested the questionnaire on 10 farm workers from a farm in another district in order to assess the flow of the questions and to what extent the questions collected the information we intended to. Information on knowledge, attitudes and practices of farm workers on agrochemicals use was collected using the pilot-tested interviewer administered questionnaire. We interviewed seven out of eleven farm owners and managers as key informants. The remainder was either not available on the day of visit or did not consent to the interview citing various reasons.

Ethical approval to conduct the research was obtained from Medical Research Council and permission from relevant government structures and farm owners. Confidentiality was assured to the participants and a signed informed consent form was used to prove that each participant had agreed to be interviewed and to have blood collected and tested.

We analyzed the data using Epi Info 2002 to generate frequencies, Odds Ratios (OR), 95% Confidence Intervals (CI) and p-values. Bivariate and multivariate analyses were conducted to determine factors that were associated with abnormal cholinesterase activity. Data from farm management were captured and analyzed by grouping together similar important points as well as noting some important contrasting information if there was any.

## Results

### Socio Demographic Characteristics of Study Participants

One hundred and forty nine (60.6%) of the respondents were males and 97 (39.4%) were females. The median age was 28 years (Q_1_=22 years, Q_3_=37 years). The median period of working with agrochemicals was 3 years (Q_1_=1 years, Q_3_=7 years). More than half (51.6%) of the respondents had at least attained secondary education. One hundred and fifty five (63%) were married, 64 (26%) were single and the remainder were either widowed or divorcees. Hundred (64.5%) of the married respondents had their spouses working as farm workers at the same farm or another farm.

### Cholinesterase Activity Results

Out of 241 (98%) respondents who consented to blood test for cholinesterase activity, 58 (24.1%) of them had abnormal cholinesterase activities of 75% or less. More females than males were affected although the difference was not statistically significant (p=0.05). The most affected age group was the 21 -30 years with 24 (41.4%) of the cases. Amongst the 58 farm workers with abnormal cholinesterase activity the most affected were sprayers (50%), followed by those who worked in previously sprayed areas 49%, loaders (31%), mixers (29%), repairers (22%), waste disposers (9%) and lastly stores managers (7%).

### Knowledge, attitudes and practices of respondents towards the use of agrochemicals

The respondents generally knew all possible routes of entry of agrochemicals into the human blood system. The routes of entry of agrochemicals were stated as nose (96%), mouth (95%), eyes (88%) and skin (83%).

One hundred and ninety eight (80.8%) knew that triangle colour codes on agrochemical containers represent degree of toxicity of the agrochemicals. Ninety eight (41.5%) knew that the most dangerous triangle colour code was purple. Those who did not know the meaning of any triangle colour code of agrochemical were more than 2 times (OR 2.03; 95% CI: 1.02 – 4.03) likely to have abnormal cholinesterase activity (19.7%, n=46) than those who were knowledgeable (Table 1).

**Table 1:** Knowledge, attitude and practices of respondents and their associations with abnormal blood test for cholinesterase activity, Kwekwe District (Zimbabwe), 2006

Variable		Cholinesterase activity ≤ 75% (N =58)	Cholinesterase activity>75% (N =183)	Odd ratio (95% CI)	p-value
Don't know colour code	Yes	17	31	2.03 (1.02-4.03)	0.04
	No	41	152		
					
Agrochemicals dangerous to health	Yes	54	164	1.56 (0.51-4.80)	0.31
	No	4	19		
					
One's duty risky due to agrochemical exposure	Yes	52	160	1.25 (0.48-3.23)	0.65
	No	6	23		
					
PPE protects from agrochemical poisoning	Yes	57	173	3.29 (0.41-26.30)	0.21
	No	1	10		
					
PPE causes discomfort	Yes	10	26	1.26 (0.56-2.79)	0.57
	No	48	157		
					
Always uses PPE when handling agrochemicals	Yes	31	98	1.00 (0.55-1.80)	0.99
	No	27	85		
					
Seldom uses PPE to protect oneself	Yes	14	28	1.76 (0.85-3.63)	0.12

Two hundred and eleven (85.8%) of the respondents knew and agreed that they were at risk of agrochemical exposures. In addition, the respondents knew that personal protective equipment (PPE) could protect them from agrochemical exposure (95.5%), periodic medical checks were beneficial (98.4%), reporting ill health to management was beneficial (91.1%) and that health education on agrochemicals was beneficial (97.6%). The perceived barriers to avoiding agrochemical exposure were the unavailability of alternative jobs (75%), management not adequately protecting workers (54%), PPE not provided (29%), fear by farm workers to report ill health to management (18%) and PPE causing discomforts and therefore could not use them (15%).

Twenty five (22.5%) reported to management when they got ill, 44 (39.6%) visited health facilities, 25 (22.5%) did nothing, whilst 6 (5.4%) treated themselves with drugs. The commonly implicated agrochemicals were Gramaxone 60 (36.6%), Tamarone 40 (24.4%), Monochrotophos 23 (14.0%), Rogor 20 (12.2%), Karate 20 (12.2%) and Dimethoate 19 (11.6%).

### Symptoms and their associations with abnormal cholinesterase activity

One hundred and eleven (45.1%) stated that they once suffered an illness that they knew or suspected to have been caused by agrochemicals. The five most common symptoms reported were headache (66.7%), cold/flu (62.2%), weakness (45.9%), dizziness (41.1%) and skin irritation (39.0%). The study did not however establish any significant association between the different forms of symptoms and abnormal cholinesterase activity (Table 2).

**Table 2:** Symptoms experienced by farm workers and their associations with abnormal blood test for cholinesterase activity, Kwekwe District (Zimbabwe), 2006

Symptom experienced		Cholinesterase activity ≤ 75% (N =58)	Cholinesterase activity>75% (N =183)	Odd ratio (95% CI)	p-value
Headache	Yes	43	120	1.51 (0.78-2.92)	0.22
	No	15	63		
					
Cold/flu	Yes	39	114	1.24 (0.67-2.32)	0.50
	No	19	69		
					
Weakness	Yes	25	87	0.84 (0.46-1.52)	0.56
	No	33	96		
					
Dizziness	Yes	25	76	1.07 (0.59-1.94)	0.83
	No	33	107		
					
Skin irritation	Yes	22	72	0.94 (0.51-1.73)	0.85
	No	36	111		
					
Sore eyes	Yes	25	71	1.20 (0.66-2.17)	0.56
	No	33	112		
					
Nausea	Yes	21	73	0.86 (0.46-1.58)	0.62
	No	37	110		
					
Abdominal pains	Yes	14	47	1.41 (0.74-2.79)	0.29
	No	39	136		
					
Fever	Yes	17	41	1.44 (0.74-2.79)	0.28
	No	41	142		
					
Blurred vision	Yes	19	38	1.86 (0.92-3.75)	0.06

### Provision of Personal Protective Equipment (PPE) and association with cholinesterase activity

One hundred and seventy six (71%) respondents were provided with at least one form of PPE. The commonest type of PPE provided was overalls (65%), gumboots (43%), gloves (22%), face masks (15%), hat 5% and goggles (4%). More males than females (130/149 vs. 44/97) were provided with PPE. Not being provided with PPE was significantly associated with abnormal cholinesterase activity (OR 2.00; 95% CI: 1.07 – 3.68). In addition, being provided with a face mask was also found to be significantly associated with abnormal cholinesterase activity (OR 2.23; 95% CI: 1.04 – 4.80) (Table 3).

**Table 3:** Personal protective equipment (PPE) provision to farmers and associations with abnormal blood test for cholinesterase activity, Kwekwe district (Zimbabwe), 2006

Type of PPE		Cholinesterase activit y ≤ 75%	Cholinesterase activity>75% (N =183)	Odd ratio (95% CI)	p-value
Gum shoes	Yes	22	81	0.77 (0.42-1.41)	0.40
	No	36	102		
Overalls	Yes	32	124	0.59 (0.32-1.07)	0.08
	No	26	59		
Face masks	Yes	13	21	2.23 (1.04-4.80)	0.04
	No	45	162		
Gloves	Yes	12	38	1.00 (0.48-2.06)	0.99
	No	46	145		
Hats	Yes	2	10	0.61 (0.13-2.90)	0.42
	No	56	173		
Goggles	Yes	2	8	0.78 (0.16-3.78)	0.55
	No	56	175		
PPE not provided	Yes	24	48	2.00 (1.07-3.68)	0.03
	No	34	135		

### Frequencies of exposure to agrochemicals and their associations with abnormal cholinesterase activity

The association of number of hours of exposure to agrochemicals with abnormal cholinesterase activity was explored using those who were working with agrochemicals for less than 2 hours as the reference population. It was established that working for more than 8 hours per day with agrochemicals was significantly associated with abnormal cholinesterase activity (OR 2.14; 95% CI: 1.17 – 3.90) (Table 4).

**Table 4:** Duration of exposure to agrochemicals for farm workers interviewed in Kwekwe District (Zimbabwe) and their associations with abnormal blood test for cholinesterase activity 2006

Type of PPE		Cholinesterase activity ≤ 75% (N =58)	Cholinesterase acti vity>75%	Odd ratio (95% CI)	p-value
2-4 hours	Yes	4	7	2.69 (0.28-1.29)	0.19
	No	10	47		
					
>4-6 hours	Yes	2	7	1.34 (0.24-7.45)	0.52
	No	10	47		
					
>6-8 hours	Yes	12	61	0.92 (0.37-2.32)	0.87
	No	10	47		
					
>8 hours	Yes	30	61	2.31 (1.03-5.20)	0.04
	No	10	47		

### Independent risk factors for abnormal cholinesterase activity

Logistic regression with the several variables under KAP, provision of PPE, health symptoms and frequency of exposure to agrochemicals based on the outcome of either having or not having abnormal cholinesterase activity was conducted using the step down regression approach and lack of knowledge on triangle colour codes and having no PPE emerged as the significant risk factors (Table 5).

**Table 5:** Independent risk factors for abnormal blood test cholinesterase activity among farm workers, Kwekwe District (Zimbabwe), 2006

Abnormal cholinesterase activity		Cholinesterase activity ≤ 75% (N =58)	Cholinesterase activity>75% (N =183)	Odd ratio (95% CI)	p-value
Don't know triangle colour codes	Yes	17	31	2.41 (1.13-2.28)	0.02
	No	41	152		
					
Having a face mask	Yes	13	21	0.69 (0.23-2.18)	0.52
	No	45	162		
					
Having no PPE	Yes	24	48	2.11 (1.11-3.99)	0.05
	No	34	135		

### Interviews with farm owners

All the farm owners agreed that their farm workers were susceptible to agrochemical exposures. They all did not have workplace health and safety programs in place. The major precaution they highlighted was the provision of PPE to the farm workers and close supervision to ensure proper use of agrochemicals. Farm owners reported making sure that farm workers were not eating or smoking during handling of agrochemicals. In addition, they restricted workers who conduct spraying to the lowest number possible in circumstances where boom spraying was not possible. The farm workers did not undergo pre-employment and routine annual medical checks to ensure that they were fit for the job they were employed for and to obtain baseline data. Responsibility for the health and safety of the farm workers was on the farm owners themselves.

The farmers stated that there were no guidelines in use to cater for the welfare of the farm workers. They also reported that they were burning all chemical waste containers and made sure that all chemicals prepared for use each day were all used. Empty chemical containers were not allowed to be taken home by farm workers for their own use. This was put in place in order to avoid chemical poisoning in the homes of the farm workers.

The major challenges highlighted by the farmers were deaths related to absenteeism from illnesses like headache, abdominal pains, weakness and the like. The nearby clinic was reported having inadequate stocks of drugs to treat diseases that most farm workers were suffering from.

## Discussion

The study revealed that the prevalence of abnormal cholinesterase activity due to agrochemical poisoning amongst the farm workers was 24.1%. The farm workers were exposed to unacceptably high levels of pesticides (i.e. those levels that cause abnormal cholinesterase activity AchE≤75%). The result is consistent with previous studies conducted in Zimbabwe before the resettlement program [6,11–13]. The findings indicate that health and safety programmes in the commercial farms in Zimbabwe are inadequate. Other studies conducted in Ethiopia have also shown that handling and storage of chemical pesticides, personal hygiene and the proper use of personal protective equipment by farm workers was below a standard [14,15] and in the Philippines poor personal hygiene and experiencing spills on farm workers bodies were risk factors [16,17].

This study also established that provision of PPE to the farm workers was lacking and where it was provided, it was inadequate. Although this study did not include the issues of appropriateness and proper usage of the PPE we cannot rule out this to be an aggravating factor. Being provided with face masks was found to be a significant risk factor. This might mean the type of PPE was either inappropriate or was not being used properly. The face masks were possibly concentrating the agrochemicals close to the nasal areas increasing risk of poisoning through inhalation or they were not being used at all because they were perceived as a hindrance to smooth air flow by the farm workers.

This study also revealed that males were less likely to have abnormal cholinesterase activity compared to females although this was not statistically significant. We established that women were mainly working in areas that would have been sprayed previously without any PPE and indirectly came into contact with residual or suspended aerosols of pesticide. Lu showed that those who re-entered recently sprayed areas had higher risk of poisoning than those who did not [16]. On the other hand more males were involved in mixing, spraying, repairing of equipment or managing the stores for pesticides where there was direct contact with pesticides and PPE provision was prioritized. Hence, males were more likely to be provided with PPE than females. This differences in the areas of work between males and females and subsequently differences in prioritization of PPE provision could be a possible explanation for the differences in cholinesterase activity between males and females.

More than 80% of our participants knew the routes of entry of pesticides in the human body and the triangle colour coding. They even knew the pesticides that were responsible for their poisoning. Contrary to this, a study in Tanzania established that majority of farm workers knew that pesticides could enter the human body and showed awareness of potential health hazards of the different pesticides used in their service areas but they did not recognize what pesticides were responsible for the poisonings [18].

However, not knowing the triangle colour code for the most dangerous agrochemicals (purple triangle) was significantly associated with abnormal cholinesterase activity. The workers may not have taken precautionary measures when handling agrochemicals due to ignorance resulting in high exposures that affected their cholinesterase activity. Other studies have shown that the preventive measures that were taken by farm workers were low, and the lower their knowledge was, the lower were the preventive measures applied [19].

Forty five percent of the participants stated that they had suffered some multiple symptoms at one point in time that they knew or suspected to have been caused by pesticide exposure. Salameh et al also established that agricultural workers have a higher prevalence of multiple symptoms, which may be due to sub-acute intoxications by pesticides that did not need hospitalization. In addition, the workers had a higher risk of having an acute intoxication due to pesticides thereby exposing them to life-threatening situations [15].

Working for more than 8 hrs per day was significantly associated with abnormal cholinesterase activity. It is expected that working more than the stipulated number of hours per day can be strenuous and fatigue cripples in thereby reducing compliance to the stipulated precautionary measures.

Spray men were affected more by chemical exposure followed by those who were working in sprayed areas. The repairers and waste disposers were the least affected. Unlike other studies, none of the types of exposures studied here was significantly associated with cholinesterase activity [6,13]. Although we could not establish after how long workers were allowed to work in the sprayed areas, this could be an important area to reckon.

Some limitations in our study were that we did not have time to look at the process of handling agrochemicals from stores to disposal due to time and resource constraints and we relied on the self reported information. Recall bias cannot be ruled out.

## Conclusion

The prevalence of abnormal cholinesterase activity among the farm workers due to chemical poisoning was high. The major exposures were spraying and working in the sprayed areas. Ignorance of the colour codes for the dangerous agrochemicals, working for more than 8 hours per day, not being provided with PPE and being provided with face masks were significantly associated with abnormal cholinesterase activity. Intensification of health education and trainings on agrochemicals handling and use, reduction of number of working hours to normal 8 hours or less, provision of adequate and proper PPE and periodic medical checks for farm workers were recommended from the findings of the study.
